# Effect of Dietary Hemp Seed on Oxidative Status in Sows during Late Gestation and Lactation and Their Offspring

**DOI:** 10.3390/ani9040194

**Published:** 2019-04-25

**Authors:** Laurentiu Mihai Palade, Mihaela Habeanu, Daniela Eliza Marin, Veronica Sanda Chedea, Gina Cecilia Pistol, Iulian Alexandru Grosu, Anca Gheorghe, Mariana Ropota, Ionelia Taranu

**Affiliations:** National Research Development Institute for Animal Biology and Nutrition, IBNA Balotesti, Calea Bucuresti nr. 1, Balotesti, 077015 Ilfov, Romania; mihaela.habeanu@ibna.ro (M.H.); daniela.marin@ibna.ro (D.E.M.); chedea.veronica@ibna.ro (V.S.C.); gina.pistol@ibna.ro (G.C.P.); grosu.iulian@ibna.ro (I.A.G.); anca.gheorghe@ibna.ro (A.G.); m.ropota@yahoo.com (M.R.); ionelia.taranu@ibna.ro (I.T.)

**Keywords:** hemp seeds, diet, PUFA, oxidative status, antioxidant enzymes

## Abstract

**Simple Summary:**

Hemp seeds are rich in polyunsaturated fatty acids as well as other bioactive compounds. Using dietary hemp seeds as late gestation and lactation supplementation for sows and early life supplementation for piglets, we found that the indicators of oxidative status were improved in both sows and offspring. Besides the significant improvement in the antioxidant defense system of the sows, our assessment of dietary intervention resulted in an array of increased antioxidative status markers for their progeny. In addition, this could be translated into increased adaptability to the upcoming weaning stage.

**Abstract:**

This study shows the antioxidant effect of a dietary hemp seed diet rich in ω-6 polyunsaturated fatty acid (PUFA) on oxidative status in sows during late gestation and lactation and their offspring. Ten pregnant sows were divided into two groups and fed either a control diet (CD) or a hemp diet (HD) containing 2% hemp seed meal for a period of 10 days before farrowing and 5% throughout the lactation period (21 d). After farrowing, 16 of their resulting piglets were divided into two groups: control group CD (eight piglets derived from control sows) and HD group (eight piglets derived from HD sows), respectively. Blood collected from sows and piglets at day 1, 7 and 21 was used for the measurement of antioxidant enzymes (catalase (CAT), superoxide dismutase (SOD), glutathione (GPx)), nitric oxide production (NO), lipid peroxidation (thiobarbituric acid reactive substances—TBARS), reactive oxygen species (ROS) generation and total antioxidant capacity (TAC) in plasma. The results showed a significant improvement in the oxidative status of sows fed HD throughout lactation compared with CD. Similarly, in piglets, HD positively influenced the activities of antioxidant enzymes, TAC and NO levels and significantly decreased lipid peroxidation in plasma until weaning, in comparison with the CD group. This study suggests the potential of hemp seed diet to improve the overall antioxidant status of the lactating sows and their progeny.

## 1. Introduction

At weaning, the changes in the piglets’ environment (separation from the sow), nutrition (switch from milk to solid feed) and regrouping (mixing with piglets from other litters) are significant and stressful [1]. Weaning induces a transient oxidative status, usually associated with low feed intake and diarrhea [2], which results in physiological and immunological disturbances, such as an increase in pro-inflammatory cytokines [1,3,4], higher concentrations of hydroperoxides (HPO) and oxidative stress index, HPO/BAP (hydroperoxide/blood antioxidant potential) [2]. Antibiotics have been widely used to control weaning-related infectious diarrhea caused primarily by an increase in *Escherichia coli* [5]. Alternative approaches, such as nutritional interventions based on bioactive compounds able to replace antibiotics, have been investigated [6,7] in pigs after the ban on antibiotics (2006). Various bioactive compounds could pass through the milk to the offspring, contributing to the development of their immune systems in early life [8,9,10]. Such interventions positively influence the health status of sows and piglets and the capacity of their offspring to better resist the stress of weaning [11]. For instance, oral administration of *Nigela sativa* (black cumin, a plant rich in bioactive constituents with antimicrobial activity) extracts, in doses equivalent to 0.0, 1.5 and 4.5 g extract kg^−1^ to weanling piglets, reduced the levels of naturally occurring E. coli in the jejunal and rectal contents [12]. The addition of *Macleaya cordata* (Wild) R. Br. extracts to feed for nursery and growing pigs decreased diarrhea occurrence and increased their weight gain [13,14]. Devi et al. (2015) showed that feeding a phytogenic additive (combination of clove, cinnamon, and fenugreek) to piglets challenged with F4+ enterotoxigenic *E. coli* (ETEC) improved their average daily gain [15]. Also, research conducted by Namkung et al. (2004), Newton et al. (2002) and Liu et al. (2016) showed that bioactive compounds from extracts of cinnamon, thyme, oregano [16] and *Sanguinaria canadensis* [14,17] inhibited gastrointestinal tract (GIT) colonization by pathogenic bacteria. The consumption of dietary linseed oil during gestation and lactation modifed the fatty acid composition of ileum, its structure and physiology, decreasing the sensitivity of the ileal epithelial barrier to mast cell degranulation in the offspring [18,19]. Increasing antioxidant nutrients in the sow diet could prevent oxidative stress by increasing antioxidant capacity [20]. These authors observed that supplementation of the diet of sows with resveratrol, a powerful antioxidant from grape, during gestation and lactation increased antioxidant enzyme levels in colostrum and milk [20]. This was benefical for piglets by increasing their antioxidant status and weight gain from birth to weaning [20]. Similarly, other bioactive compounds from soy, ginger, grape, etc., alleviate oxidative stress (increasing plasma antioxidants, decreasing pro-inflammatory response) and improve the performance of sows and piglets [21,22,23]. Both the in vivo and in vitro evidence showed the effect of such compounds in preventing inflammation and oxidative stess. The study of Lin et al. (2016) reported that docosahexaenoic acid (DHA), an ω-3 polyunsaturated fatty acid (PUFA), attenuated LPS-induced inflammatory responses in porcine mammary epithelial cells through modulation of the TLR4 pathway [24]. Also, Horn et al. (2017) showed that garlic compounds mitigate oxidant-induced cellular dysfunction, modulate innate immune function, and stimulate the proliferation of lymphocytes [6].

Hemp *(Cannabis sativa* L.) is one of the many plants rich in bioactive compounds that have been exploited for health food and medicinal purposes [25,26,27]. Recently, interest in hemp has been largely focused on seeds, which are nutritionally beneficial for both humans and animals [28]. Hemp seeds (HS) are a great source of phytochemicals, containing around 30% fats and 25% proteins as well as fiber, cannabinoids, vitamins and minerals [29,30,31]. They also contain polyphenols (caffeic acid, quercetin and luteolin) [31], lignans [32], terpenoids [27], as well as protein hydrolysates, which have been shown to reduce oxidative stress factors in hypertensive rats [33]. They are known especially for containing polyunsaturated fatty acids (PUFAs), mainly ω-6 and ω-3, which are active molecules studied for their anti-inflammatory action, immune and oxidative stress modulation [29,34,35,36]. Whole hemp seeds oil contains 75–80% polyunsaturated fatty acids (PUFA), including 60% linoleic acid (LA) and 17 to 19% α-linolenic acid (ALA) [37,38,39]. 

Few studies have looked at the effects of hemp seeds on oxidative status and health in the diets of pigs and other animals. However, Prociuk et al. (2006) have shown the beneficial effects of dietary hemp seeds in hypercholesterolemic rabbits following cardiac ischemia/reperfusion injury [40], which can lead to oxidative stress [41]. A direct anti-inflammatory effect (decreased release of pro-inflammatory mediators by mast cells, attenuated inflammatory pain and decreased generation of NO) caused by cannabinoids (chemical constituents of hemp) was described in animal models such as guinea pigs [42] and mice [43,44,45]. Similarly, Hong et al. (2015) observed that hemp seed effectively inhibited H_2_O_2_-mediated oxidative stress by increasing the gene expression of antioxidant enzymes in HepG2 cells [46]. It is generally accepted that, not only during delivery, but also during late gestation and early lactation, the sows show elevated oxidative stress [47,48]. Lipid peroxidation increases, while the antioxidant status is weakened, leading to oxidative stress that can affect the fetus [49,50]. Oxidative stress diminishes the ability of sows to produce milk, which in turn has a negative influence on the growth of their piglets [48]. Also, under persistent stressful conditions, the formation of large numbers of free radicals and reactive oxygen species (ROS) occurs and could negatively affect the endogenous antioxidant system [51]. During these periods, low intake of antioxidant nutrients may be one of the main causes of systemic oxidative stress in sows [52,53]. At this point, a nutritional intervention based on dietary antioxidant enrichment is regarded as a viable solution. For example, previous results obtained by Mahan and Peters (2004) and Umesiobi (2009) with antioxidants such as vitamin E and selenium showed a reduction of oxidative stress in sows [54,55]. In line with this, Zhan et al. (2011) reported that the intake of selenomethionine during gestation and lactation improved antioxidant status and decreased lipid peroxidation levels in the serum of sows [56]. 

Besides the large numbers of free radicals and reactive oxygen species, oxidative stress leads to the imbalanced generation of important molecules in the body [51]. As such, nitric oxide (NO) is generated through the L-arginine–nitric oxide pathway, involving both the inducible nitric oxide synthase (iNOS) and the endothelial nitric oxide synthase (eNOS) [57,58]. The uncoupling of this pathway results in the decreased formation of NO [58], which can be attributed to elevated oxidative stress [59]. Very importantly, NO is also generated as a host defense response against intracellular pathogens [57]. Increased production of NO (above the physiological levels) has been documented in macrophages and neutrophils with antimicrobial and cytostatic properties in animal models [60,61].

With regard to the health status of suckling piglets, several studies have found beneficial effects. Thus, the report of Xie et al. (2015) showed that piglets derived from sows fed a chitosan oligosaccharide supplemented diet during late gestation and lactation had reduced hypoglycemia and an increased growth rate [62]. Similarly, Yao et al. (2012) observed an improvement in the performance and immune status of piglets when the dietary ratio of ω-6:ω-3 PUFAs in lactating sows was 9:1 [63]. 

The present study was designed to assess the effects of dietary hemp seed inclusion on oxidative status in the plasma of sows and resulting progeny. Hemp seed was included in the diet of sows from late gestation through lactation as an early life intervention for piglets to better deal with stress during weaning. Antioxidant enzyme activities, total antioxidant capacity, nitric oxide, ROS and TBARS concentration were measured at individual time points. 

## 2. Materials and Methods

### 2.1. Animals and Treatments

Animals were cared for in accordance with the Romanian Law 206/2004 and the EU Council Directive 98/58/EC for the handling and protection of animals used for experimental purposes. The study protocol was approved by the Ethical Committee of the National Research-Development Institute for Animal Nutrition and Biology, Balotesti, Romania (Ethical Committee no. 52/2014). All animals remained healthy during the experimental period and no veterinary drugs were used.

This study is part of a wider experiment (the same design as the study of Habeanu et al. 2018 [64]), where a total of 123 piglets were born, of which 11 were stillborn and 16 died throughout the experimental period. Ten multiparous gestation TOPIGS-40 hybrid (♀ Large White × Hybrid (Large White × Pietrain) × ♂ Talent, mainly Duroc) sows were allocated to two experimental groups (5 sows/group). The animals were housed in pens and fed a control diet (CD) and a control diet with 2% ground hemp seed (HD) for the last 10 days of gestation. After farrowing (resulting in an average litter size of 10 piglets/sow) the content of hemp was increased to 5% in the experimental diet, which was given ad libitum to lactating sows for 21 days. The Romanian Jubileu hemp seed variety (hemp variety for seed/grain production), delivered by the Agricultural Research-Development Base Secuieni-SCDA Secuieni, was used. 

After farrowing, 16 of the resulting piglets were divided into two groups: control group CD (8 piglets derived from control sows) and HD group (8 piglets derived from HD sows), respectively. Starting on day 10 after birth, piglets had ad libitum access to the control or hemp diets (1.5% hemp seed inclusion), they were studied until day 21 (weaning day).

Pelleted diets were formulated to be isoproteic and isocaloric and to meet all nutritional requirements specific to TOPIGS hybrid [65] and at each life stage as indicated by the National Research Council (NRC—2012) [66], with the following chemical composition: pregnant sows (crude protein, 14.7%; fat, 5%; crude fiber, 6.4% and 1.2 metabolizable energy (ME—MJ/kg); lactating sows: (crude protein, 16.9%; fat, 4.5%; crude fiber, 6.5% and 1.3 ME (MJ/kg) and piglets (crude protein, 19.6%; fat, 2.8%; crude fiber, 4.1% and 1.4 ME (MJ/kg) as described by Hăbeanu et al. (2018) [64]. The percent of hemp inclusion in the diet was according to EFSA Opinion (2011), which clearly states the maximum incorporation rates for hemp seed and hemp seed cake (2–5% inclusion) [67].

The gross chemical composition of hemp and experimental feed was analyzed according to the Romanian Standardization Association (ASRO) methods: dry matter (SR ISO 6496:2001), crude protein (SR EN ISO 9583-2:2009), fat (SR EN ISO 6492:2001), crude fiber (SR EN ISO 6865:2002) and ash (SR EN ISO 2171:2010) [68]. Hemp seed and experimental compound feed diets were evaluated for the total polyphenol content (TPC), antiradical activity (A_AR_) using the Folin–Ciocalteu and 2,2-diphenyl-1-picrylhydrazyl (DPPH) methods. The polyphenol profile was determined by high performance liquid chromatography coupled with mass spectrometry (LC–MS). The extraction procedure involved a maceration step in a mixture of methanol-acetone-water (M:A:W, 7:7:6) for 24 hours at 37 °C under continuous shaking according to the method of Teh et al. (2014) [31].

### 2.2. Blood Sample Collection 

Blood samples were aseptically collected by venipuncture (fasting animals) from the jugular vein from sows 10 days before farrowing (initial time point), immediately after farrowing (day 1) and on day 7 and 21 of lactation as well as from piglets on day 1, 7 and 21 after birth. Blood was collected into 9-mL Vacutainer tubes containing 143 U/mL of lithium heparin (Vacutest^®^, Arzergrande, Italy) and processed within one hour after collection by centrifugation at 2500 rpm, for 10 min, at 4 °C (Multifuge 3L-R, Heraeus, Hanau, Germany). Plasma samples were frozen immediately after collection and stored at −80 °C until analysis and were used for the measurement of antioxidant enzymes catalase (CAT), superoxide dismutase (SOD), glutathione peroxidase (GPx), total antioxidant capacity (TAC), nitric oxide production (NO), lipid peroxidation ((tpc) and reactive oxygen species (ROS).

### 2.3. Determination of Bioactive Compounds in Hemp Seed and Diets

#### 2.3.1. Determination of total Polyphenol Content and Antiradical Activity

The TPC from hemp seed and experimental feed compounds was determined using the Folin–Ciocalteu method previously described by Arnous et al. (2002). It is a colorimetric in vitro assay of polyphenolic antioxidants, which uses gallic acid (known polyphenol) as the reference standard. A volume of 5 µL of undiluted sample was mixed with 795 µL of distilled water and 50 µL of Folin–Ciocalteu reagent; after exactly 1 min, 150 µL of 20% Na_2_CO_3_ was added and the mixture was incubated in the dark at room temperature for 2 h; the absorbance was read at 765 nm. Results were expressed as mg gallic acid equivalents (GAE) per 100 g of sample [69]. 

The antiradical activity (A_AR_) was measured using the stable radical DPPH as described by Arnous et al. (2002). Briefly, an aliquot of 25 μL of diluted sample was added to 975 μL of DPPH solution (60 μM in methanol) and vortexed, the absorbance was read at 515 nm at *t* = 0 and *t* = 30 min. Results were expressed as µM Trolox equivalents (TE) [69]. 

#### 2.3.2. Determination of Polyphenol and Cannabinoid Profile (LC–MS)

The chromatographic measurements were performed using a complete HPLC Agilent 1200 system using a C18 Eclipse XDB column (4.6 × 150 mm, 5 μm). The HPLC system was coupled to a mass spectroscopy detector, Agilent 6110 single quadrupole (Agilent Technologies, CA, USA). The analytical mobile phase consisted of 0.1% acetic acid in water (solvent A) and 0.1% acetic acid in acetonitrile (solvent B). The compounds were separated using gradient elution with 0.5 mL/min flow at 25 °C for 30 min as follows: 5% solvent B for 2 min, increased to 40% until min 18 min, increased to 90% until 20 min, maintained at 90% for 4 min, decreased to 5% until 25 min, followed by another 5 min at 5 % for reconditioning. Chromatograms were recorded at 280 and 340 nm. Mass spectra were acquired in the positive ESI mode: 3000 V, 300 °C, 8 L/min nitrogen flow, m/z:100−1000, full-scan. Data acquisition was achieved using the ChemStation software (Agilent Technologies). The results were expressed as µg catechin equivalents (CE) /mL extract.

#### 2.3.3. Determination of Polyunsaturated Fatty Acid (PUFA) Profiles

PUFAs were measured in hemp seeds and diets as fatty acid methyl esters (FAME) by gas chromatography using a Perkin Elmer-Clarus 500 gas chromatograph, fitted with a Flame Ionization Detector and capillary separation column with high polar stationary phase Agilent J&WGC Columns, method described by Hăbeanu et al. (2014) [70]. 

### 2.4. Measurement of Antioxidant Enzyme Activities (CAT, SOD, Gpx) in Plasma 

Catalase (CAT), superoxide dismutase (SOD) and glutathione peroxidase (GPx) activities were measured using standardized kits (Cayman Chemical) according to the manufacturer’s instructions. The absorbance was measured using a Tecan microplate reader (Tecan, SunRise, Austria). Catalase activity was measured at 540 nm and the results were expressed as nmol/min/mL. The method is based on the reaction of an enzyme with methanol in the presence of an optimal H_2_O_2_ concentration. The produced formaldehyde is measured colorimetrically with 4-amino-3-hydrazino-5-mercapto-1,2,4-triazole (Purpald) as a chromogen. “Purpald specifically forms a bicyclic heterocycle with aldehydes, which upon oxidation, changes from colourless to purple” [71]. One unit was defined as the amount of enzyme that will cause the formation of 1.0 nmol of formaldehyde per minute at 25 °C. SOD activity was measured at 440–460 nm and the results were expressed as U/mL. The assay allows the measurement of all three types of SOD enzymes and uses tetrazolium salt for detection of superoxide radicals generated by hypoxanthine and xanthine oxidase. One unit was defined as the amount of enzyme needed to exhibit 50% dismutation of the superoxide radical. Superoxide dismutase activity is standardized using the cytochrome c and xanthine oxidase coupled assay. Glutathione peroxidase activity was indirectly measured by a coupled reaction with glutathione reductase (GR) through which the oxidation of NADPH to NADP+ is translated by a decrease in the absorbance measured every minute at 340 nm (for 10 min) and results were expressed as nmol/min/mL. One unit was defined as the amount of enzyme that will cause the oxidation of 1.0 nmol of NADPH to NADP^+^ per minute at 25 °C. The CAT assay kit intra-assay coefficient of variation (CV) was 3.8% and the inter-assay CV was 9.9%. We obtained an intra-assay CV of 8.43% and an inter-assay CV of 13.02%. The SOD assay kit intra-assay CV was 3.2% and the inter-assay CV was 3.7%. We obtained an intra-assay CV of 9.31% and an inter-assay CV of 17.08%. The GPx assay kit intra-assay CV was 5.7% and the inter-assay CV was 7.2%. We obtained an intra-assay CV of 10.47% and an inter-assay CV of 15.02%.

### 2.5. Measurement of Plasma Lipid Peroxidation (TBARS)

Plasma lipid peroxidation was analyzed as previously described by Ohkawa et al. (1979) [72]. The TBARS assay principle is based on the measurement of malondialdehyde (MDA) present in the sample as a by-product of lipid peroxidation. Briefly, plasma was mixed with deionized water, 0.5 N HCl and thiobarbituric acid (TBA), and incubated at 95 °C for 15 min. The measurement was obtained in fluorescence mode (exc. 515 nm; em. 548 nm) using a microplate reader (Tecan Infinite M200). The results were expressed as nmol MDA/mL of plasma using 1,1,3,3,-tetramethoxypropane (TMP) as standard. We obtained an intra-assay CV of 7.96% and an inter-assay CV of 16.93%.

### 2.6. Measurement of Plasma Total Antioxidant Capacity (TAC)

Total antioxidant capacity in plasma was assayed using a QuantiChrom kit (BioAssay Systems, Hayward, CA, USA). The assay measures total antioxidant capacity in which Cu^2+^ is reduced by antioxidant to Cu^+^. The resulting Cu^+^ specifically forms a colored complex with a dye reagent. The color intensity at 570 nm is proportional to TAC in the sample. Briefly, 20 µL of undiluted plasma or Trolox standard solution along with 100 µL working reagent was added to a 96-well microplate, mixed by tapping and incubated at room temperature for 10 min according to the manufacturer’s recommendations. The absorbance of the reaction was read at 570 nm using a microplate reader (Tecan Infinite M200) [73]. Results (TAC) were expressed as µM Trolox equivalents. We obtained an intra-assay CV of 9.93% and an inter-assay CV of 15.91%.

### 2.7. Measurement of Plasma Nitric Oxide Production (NO)

Nitric oxide (NO) levels in plasma were evaluated using the Nitrate/Nitrite Colorimetric Assay Kit (Cayman Chemical), according to the manufacturer’s instructions. The first step of the assay is the conversion of nitrate to nitrite using nitrate reductase. Further, the addition of the Griess Reagents converts nitrite into a purple azo compound. The absorbance measurement of this azo chromophore accurately determines NO_2_^−^ concentration. Briefly, 80 µL of appropriately diluted sample (plasma) was mixed with 10 µL of enzyme cofactor mixture and 10 µL of nitrate reductase mixture. Then, the plate was covered and incubated for 3 h at room temperature. Subsequently, 50 µL of Griess Reagent R1 followed immediately by 50 µL of Griess Reagent R2 was added to each of the wells. The color was allowed to develop for 10 min at room temperature. Absorbance was read at 550 nm using a microplate reader (Tecan Infinite M200). Results (NO) were expressed based on a NaNO_2_ standard curve with a concentration range of 0 to 100 µM [74]. The NO assay kit intra-assay coefficient of variation (CV) was 2.7% and the inter-assay CV was 3.4%. We obtained an intra-assay CV of 11.39% and an inter-assay CV of 20.44%.

### 2.8. Measurement of Plasma Reactive Oxygen Species (ROS)

The determination of ROS in plasma was evaluated using 20 μM 2′,7′- dichlorodihydrofluorescein diacetate (DCF-DA) which undergoes hydrolyzation by cellular esterases to produce DCF (2′,7′ dichlorofluorescein). The reaction of DCF with ROS forms fluorescent DCF. Fluorescence signal was measured after 4 h in 96-well black plates at an excitation wavelength of 485 nm and emission wavelength of 528 nm using a microplate reader (Tecan Infinite M200). Results are expressed as fluorescence arbitrary units (a.u.). We obtained an intra-assay CV of 8.36% and an inter-assay CV of 11.83%.

### 2.9. Statistical Analysis

All data are expressed as mean ± standard error of the mean (SEM). All the results were submitted to SAS University Edition software (SAS Analytics, USA) and JMP 11 Statistical Discovery^TM^ from SAS. Repeated measurements analysis (repeated ANOVA) was performed to investigate the statistical differences between groups for all analyzed parameters employing the Restricted Maximum Likelihood (REML) method. The repeated measures design reduces the variance of estimates of treatment-effects, allowing statistical inference to be made with fewer data. The model effects were diet (CD or HD), time (gestation, day 1, day 7 and day 21) and their interaction (D × T) in order to evaluate the overall treatment effect. Each sow was considered an experimental unit. For piglets, the statistical model included litter as a random effect. Values of *p* < 0.05 were considered significant. 

## 3. Results

### 3.1. Bioactive Compounds

#### 3.1.1. Total Polyphenol Content and Antiradical Activity 

The total polyphenol content (TPC) and the antiradical activity (A_AR_) was measured in both the control and hemp diets as well as in hemp seed. The results from Table 1 showed no difference in TPC and antiradical activity between the control diet and hemp diet (5% inclusion) (Table 1).

In addition, the results of the LC–MS analysis, given as mg cathechin equivalents/100 g feed, revealed the presence of several polyphenolic compounds (ferulic acid dehydrotrimer, caffeoyl quinic acid, daidzein glucoside, genistein glucoside, ferulic acid and genistein malonylglucoside) in similar amounts in both the control and the hemp seed diets (5% inclusion).

#### 3.1.2. Polyunsaturated Fatty Acid Profiles

The polyunsaturated fatty acid (PUFA) profiles were previously reported by Hăbeanu et al. (2018) [64]. PUFAs represented 72.58% of total FAMEs (fatty acid methyl esters) in HS, with 55.28% ω-6 and 17.3% ω-3. Linoleic acid (LA) was the most abundant ω-6 FA (53.79%), whereas alpha-linolenic acid (ALA) was the predominant ω-3 FA (17.06%). Oleic acid (ω-9 MUFA) was also present with a concentration of 14.46% (of total MUFA 14.56%). For example, the diet with 5% HS in sows translated into a 53% increase in ω-3 PUFA compared to the control diet, as well as a 34.32% decrease of ω-6:ω-3 ratio in favor of HD. The piglets’ feed (1.5% HS inclusion) contained 5.73% ω-3 PUFA and 51.01% ω-6 PUFA, which shows a lower ω-6:ω-3 ratio compared with the control (8.9% vs 13.55%) [64].

#### 3.1.3. Cannabinoid Profile

Similar to the profile of polyphenols, cannabinoids were analyzed by LC–MS and the results were expressed as mg cathechin equivalents/100 g feed. The profile obtained for HD (5% inclusion) included several cannabinoids such as cannabigerol (CBG—66.09 mg CE/100g feed), cannabigerolic acid (CBGA-46.85 mg CE/100g feed), ∆9-tetrahydrocannabinol (∆9-THC-48.16 mg CE/100g feed) and ∆9-tetrahydrocannabinolic acid (∆9-THCA–28.65 mg CE/100g feed), which accounted for a total of 164.4 mg CE/100 g of feed. 

### 3.2. Animal Performance

Animal performance (average daily feed intake and average weight gain) was described in detailed by Habeanu et al. (2018) [64]. Briefly, there were no significant differences in the daily feed intake of sows either in gestation or in lactation (2.5 kg/day/sow in gestation and 5.16 kg/day/lactating control sow vs. 5.57 kg/day/HS lactating sow). The average daily gain (ADG) of piglets at 21 days was 0.169 kg for the CD group compared to 0.189 kg for the HD group.

### 3.3. Effect of Hemp Dietary Intervention on Oxidative Status in Sows

#### 3.3.1. Reactive Oxygen Species, Lipid Peroxidation, Total Antioxidant Capacity and Nitric Oxide

**Figure 1:**
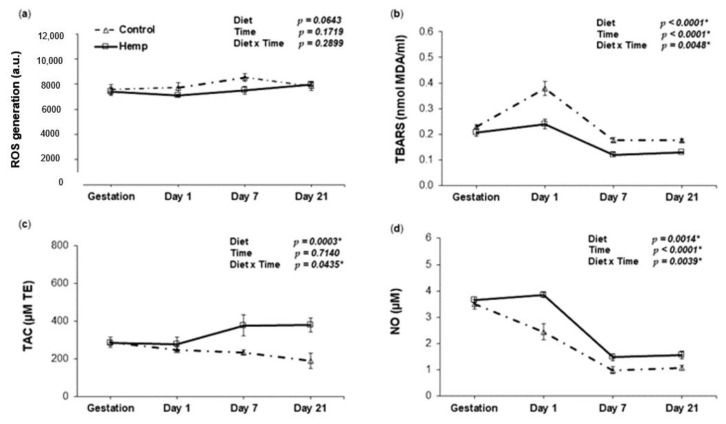
The effect of HD on oxidative status in the plasma of sows throughout the lactation period. (**a**) Relationship between reactive oxygen species (ROS) and day of lactation in the plasma of sows fed a control (Δ symbol) or a hemp seed diet (□ symbol); (**b**) Relationship between lipid peroxidation (TBARS) and day of lactation in the plasma of sows fed a control (Δ symbol) or a hemp seed diet (□ symbol); (**c**) Relationship between total antioxidant activity (TAC) and day of lactation in the plasma of sows fed a control (Δ symbol) or a hemp seed diet (□ symbol); (**d**) Relationship between NO and day of lactation in the plasma of sows fed a control (Δ symbol) or a hemp seed diet (□ symbol). The *p*-values for the effects of treatment (diet), time and diet-time interaction are shown. Data are means ± SEM (*n* = 5). Asterisk (*) indicates significance (*p* < 0.05 *).

Main effects of diet (D), time (T) and their interaction (D × T) for the markers associated with oxidative status in lactating sows were assayed. Reactive oxygen species (ROS) in the plasma of sows fed HD diet did not decrease (*p* = 0.15) immediately after farrowing (day 1), but decreased significantly on day 7 (Figure 1a). This decrease was not maintained until the end of the period (day 21). Repeated measurements analysis revealed a tendency to lower ROS generation throughout the study period (D, *p* = 0.06). Similarly, HD reduced the plasma TBARS levels in sows with significant main effects of D (*p* < 0.0001 *), T (*p* < 0.0001 *) and D × T (*p* = 0.0048 *) during the lactation period as shown in Figure 1b.

At the same time, the plasma total antioxidant capacity (TAC) increased significantly both at day 7 and day 21 (Figure 1c). The improvement in TAC by HD over the control was displayed by the significance levels of D (*p* = 0.0003 *) and D × T (*p* = 0.04 *). The hemp diet increased NO synthesis in HD sows compared to CD sows during lactation (Figure 1d), reflected by the effects of D (*p* = 0.0014 *) and time (T, *p* < 0.0001 *). The D × T interaction (*p* = 0.0039 *) shows that the pattern over time was different for the two diets, with a delayed decrease in NO in HD sows. 

#### 3.3.2. Antioxidant Enzyme Activities 

**Figure 2:**
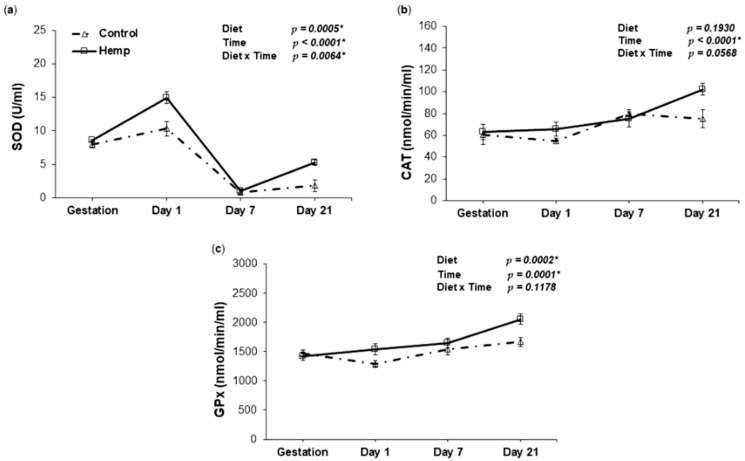
The effect of HD on antioxidant enzyme activities in the plasma of sows throughout the lactation period. (**a**) Relationship between superoxide dismutase (SOD) activity and day of lactation in the plasma of sows fed a control (Δ symbol) or a hemp seed diet (□ symbol); (**b**) Relationship between catalase (CAT) activity and day of lactation in the plasma of sows fed a control (Δ symbol) or a hemp seed diet (□ symbol); (**c**) Relationship between glutathione peroxidase (GPx) activity and day of lactation in the plasma of sows fed a control (Δ symbol) or a hemp seed diet (□ symbol). The *p*-values for the effects of treatment (diet), time and diet-time interaction are shown. Data are means ± SEM (*n* = 5). Asterisk (*) indicates significance (*p* < 0.05 *).

The effect of HD on antioxidant enzymes as key components of the antioxidant defense system (CAT, SOD and GPx) was assayed in the plasma of sows from late gestation through lactation day 21. Our results (Figure 2) show a significant increase in SOD activity on days 1 and 21 in sows fed HD. Although SOD activity decreased over time for both the hemp and control diets (T, *p* < 0.0001 *), it registered elevated levels in HD sows compared to the control fed sows, as revealed by the main effect of D (*p* = 0.0005 *) (Figure 2a). The plasma of sows also showed an increase in CAT activity for both diets (T, *p* < 0.0001 *), whereas only a trend was noticed for the D × T interaction (*p* > 0.05) (Figure 2b). Similarly, repeated measurements analysis revealed an increase in GPx activity over time in both diets (T, *p* = 0.0001 *), but the increase for HD was greater than the increase for CD (D, *p* = 0.0002 *) (Figure 2c). 

### 3.4. Effect of Hemp Dietary Intervention on Oxidative Status In Piglets

#### 3.4.1. Reactive Oxygen Species, Lipid Peroxidation, Total Antioxidant Capacity and Nitric Oxide 

**Figure 3:**
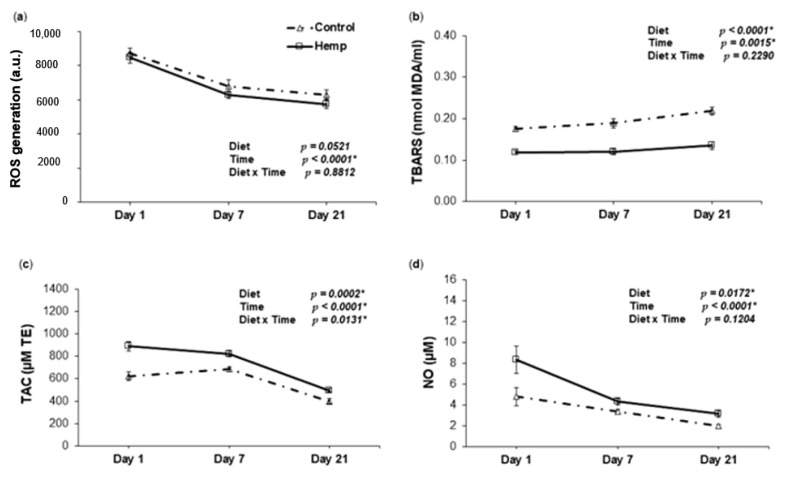
The effect of HD on oxidative status in the plasma of piglets throughout the lactation period. (**a**) Relationship between reactive oxygen species (ROS) and day of lactation in the plasma of piglets fed a control (Δ symbol) or a hemp seed diet (□ symbol); (**b**) Relationship between TBARS and day of lactation in the plasma of piglets fed a control (Δ symbol) or a hemp seed diet (□ symbol); (**c**) Relationship between total antioxidant capacity (TAC) and day of lactation in the plasma of piglets fed a control (Δ symbol) or a hemp seed diet (□ symbol); (**d**) Relationship between NO and day of lactation in the plasma of piglets fed a control (Δ symbol) or a hemp seed diet (□ symbol). The *p*-values for the effects of treatment (diet), time and diet-time interaction are shown. Data are means ± SEM (*n* = 8). Asterisk (*) indicates significance (*p* < 0.05 *).

Reactive oxygen species (ROS) levels decreased in time in the plasma of piglets irrespective of the treatment. The decreasing trend during lactation reflects a significant effect of time for both HD and CD piglets (*p* < 0.0001 *), with a tendency for lower levels of ROS in HD piglets (D, *p* > 0.05) (Figure 3a). The plasma TBARS levels of piglets belonging to HD sows decreased during lactation below that of the control, but increased over time for both treatments (T, *p* = 0.0015 *) (Figure 3b). 

Total antioxidant capacity (TAC) levels were higher overall in HD piglets compared to the control (D, *p* = 0.0002 *), but decreased over time. On the contrary, the decrease in CD piglets was observed only from day 7 to 21 (Figure 3c). 

Similarly, piglets derived from sows fed HD during the last 10 days of gestation had significantly higher plasma nitric oxide concentration compared to the control during the sucking period. NO levels decreased over time for both HD and CD piglets (Figure 3d). 

#### 3.4.2. Antioxidant Enzyme Activities 

**Figure 4:**
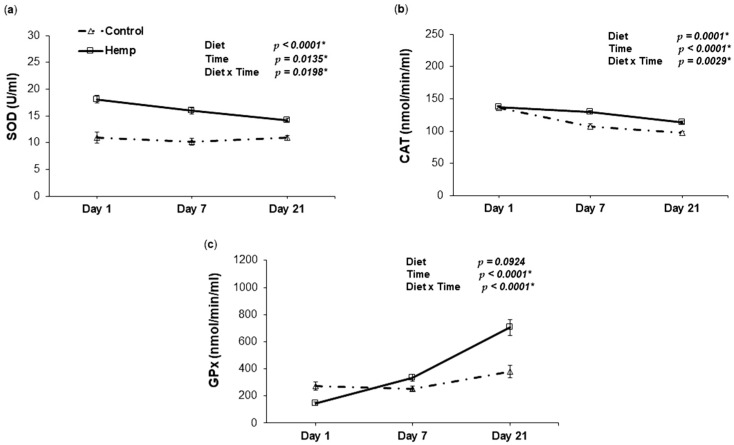
The effect of HD on antioxidant enzymes activities in the plasma of piglets throughout the lactation period. (**a**) Relationship between SOD activity and day of lactation in the plasma of piglets fed a control (Δ symbol) or a hemp seed diet (□ symbol); (**b**) Relationship between CAT activity and day of lactation in the plasma of piglets fed a control (Δ symbol) or a hemp seed diet (□ symbol); (**c**) Relationship between GPx activity and day of lactation in the plasma of piglets fed a control (Δ symbol) or a hemp seed diet (□ symbol). The *p*-values for the effects of treatment (diet), time and diet–time interaction are shown. Data are means ± SEM (*n* = 8). Asterisk (*) indicates significance (*p* < 0.05 *).

The activity of SOD in the plasma of piglets derived from sows fed HD during the last 10 days of gestation registered higher levels than for CD piglets, which is confirmed by the main effect of D (*p* < 0.0001 *) and decreased over time, whereas SOD activity for CD piglets did not change over time (D × T, *p* = 0.0198 *) (Figure 4a). The activity of CAT was not different on day 1, but decreased more in CD than in HD piglets throughout the period until weaning (Figure 4b). On day 1, GPx activity was lower for HD than for CD piglets. However, GPx activity in HD piglets increased much more over time than in CD piglets, resulting in higher activity on days 7 and 21 in HD piglets (Figure 4c).

## 4. Discussion

Current challenges in livestock production refer to the increasing competition for natural resources, as well as to the development of strategies focused on replacing antibiotics as dietary growth promoters [75]. After the ban of antibiotics (2006), nutritional interventions using plant by-products as feed additives rich in bioactive compounds have been intensively investigated for their beneficial health effects and therapeutic potential [76,77]. In this regard, there is a wide variety of natural sources which are investigated for their health-promoting properties. Hemp (*Cannabis sativa* L.) is an example and, besides its other industrial uses, it is a good source of PUFAs, polyphenols, tocopherols, proteins, vitamins, minerals, etc. [27]. 

In this study, we showed that the inclusion of hemp seed in the diet of late gestation sows and during the suckling period of piglets influences the systemic oxidative status of both sows and offspring by increasing antioxidant enzyme activities, total antioxidant capacity and nitric oxide synthesis, and by decreasing the lipid peroxidation in plasma. The hemp seed used in our work has fallen into the already mentioned [37,38,39] levels for fat, 53.79% of linoleic acid (18:2n-6) and 17.06% linolenic acid (18:3n-3) with a ω-6: ω-3 ratio of 3.19 [64]. The inclusion of this hemp seed in the diets of sows during late gestation and lactation decreased by 34.32% the dietary ω-6: ω-3 ratio, the key index for balanced synthesis of eicosanoids in the body [78,79], compared to the control diet. The concentration of cannabinoid compounds was also in the range reported by other studies [25,45,80]. By contrast, the LC–MS method used in our study detected only negligible polyphenol concentrations.

In the present study, we have identified that the effects of the two diets are different in terms of lipid peroxidation measured in the plasma of sows, as indicated in Figure 1. Feeding hemp over the last 10 days of gestation has proven efficacy in the fact that at farrowing (day 1), the lipid peroxidation (TBARS) level decreased and the NO concentration increased in sows from the HD group compared to the control group. The beneficial effect on these parameters was maintained also throughout lactation. The positive effect of HD on antioxidant capacity (TAC) was manifested later, differences against the control being observed only after day 7. This is reflected by the D–T interaction (*p* = 0.0048 *), suggesting the efficacy of the diet (D) rich in active compounds and the time of utilization (T). By contrast, there were no differences in ROS between the two groups. A possible explanation might be that TBARS measures the products in the terminal phase of lipid peroxidation, while ROS are produced as intermediary products and could appear in both the initiation and the propagation phases [81,82]. Hence, it is possible that ROS could have been generated in similar concentrations in the two groups (control and hemp), but the process of lipid peroxidation would have been inhibited (slowed down) due to the increased activity of the antioxidant enzymes in sows fed HD. This effect could also be perceived in conjunction with TAC, which rises over time in HD sows, but falls over time in CD sows. The significant D–T interaction (*p* = 0.04 *) observed for TAC indicates that the response to diet over time is different between the two groups. Taken together, in our study, we assume from the observed changes of TBARS, TAC and NO levels in plasma that HD exerts a preventive effect towards the action of free radicals. Also, Tan et al. (2015) found that dietary oregano essential oil, known for its antioxidant activity, significantly reduced serum lipid peroxidation in sows on day 1 of lactation [47]. 

Our study indicated that HS provided a higher level of PUFAs to the experimental diet. PUFAs are known for their capacity to increase the gene expression and activity of several antioxidant/detoxifying enzymes [83]. Indeed, we found a significant increase in plasma antioxidant status in sows and piglets receiving HD which suggests that PUFAs contributed to the improvement in antioxidant markers. Most importantly, SOD shows different trends over time for the two diets in sows, indicated by the significant D–T interaction (*p* = 0.0064 *). As in the case of TBARS and NO, feeding HS during gestation contributed to an increased level in SOD activity at farrowing (day 1) compared to the control. A decrease in SOD activity was observed at day 7 irrespective of the diet, but dietary HS was able to increase its activity on day 21 when compared to CD. By contrast, the effect of HD on CAT activity was noted only at the end of the lactation period (day 21). Although HD significantly increased GPx activity during the lactation period, the D × T interaction was not significant. Research involving PUFA highlighted these beneficial effects. For example, investigating the influence of PUFAs as a dietary intervention during gestation and lactation, Shen et al. (2015) observed that sows fed a fish oil supplemented diet rich in PUFAs during late pregnancy and lactation showed an overall positive increase in plasma total antioxidant capacity and activities of GPx and SOD [84]. In a similar manner, Hu et al., 2015, reported that dietary supplementation with glycitein (soy isoflavone) during late pregnancy and lactation elevated the antioxidative indices (SOD, TAC, CAT and GPx), decreased lipid peroxidation levels in the plasma and milk of sows, improved milk composition, and enhanced the growth performance of sucking piglets [22]. As observed by Lluís et al. (2013) in a rat model, eicosapentaenoic acid (EPA) and docosahexaenoic acid (DHA), which are PUFAs, appear to exert protective effects by improving the SOD and GPx levels in the plasma of the ω-3 PUFA supplemented group [85]. It has been reported that the molecular mechanism by which PUFAs induced the stimulation of antioxidant status is related to the activation of Nrf2 and the inhibition of NFkB, two nuclear factors responsible for protection against oxidative stress and induction of inflammation [83]. All these examples suggest that the inclusion of antioxidant-rich sources in sow diet during gestation could be considered as an efficient early nutrition intervention with a positive effect on offspring during lactation (improved antioxidant status). Our short-term nutritional intervention also pointed out increased TAC levels and antioxidant enzyme activities during the last stage of lactation. In the same register, Su et al. (2017) reported that the systemic oxidative status was affected by antioxidant supplementation in sows across lactation, with the total SOD activity standing out in terms of decline during this period [53].

Polyunsaturated fatty acids (PUFAs) appear to interact with the metabolism of the L-arginine–nitric oxide (NO) system [86] and, in our experiment, we observed a significant effect of HD on NO synthesis throughout lactation. NO levels were higher for the HD diet (D), decreased over time for both diets (T), but the decreases were not parallel (D × T). The fact that, on day 1, there was a significant improvement in NO levels for HD sows shows that the 2% dietary hemp seed inclusion for the 10 days of gestation should be perceived as an efficient dietary intervention. Nitric oxide is important in regulating blood flow to various organs including mammary glands by relaxing the smooth muscle tissue [87]. Several studies reported that dietary arginine supplementation enhanced mammary plasma flow by increasing nitric oxide in endothelial cells causing vasodilatation and increased mammary nutrient uptake [48,87]. 

Besides PUFAs, the potential effects of cannabinoids are not to be overlooked. For instance, Borreli et al. (2013) investigated the effect of a hemp extract containing cannabigerol (CBG), a non-psychotropic cannabis-derived cannabinoid, in a murine model of colitis [88]. Their assessment showed that CBG attenuated the colitis by improving SOD activity and NO production, and indicated normalized levels (towards the control) of interleukin-1β, interleukin-10 and interferon-γ, as well as reduced ROS formation in intestinal epithelial cells [88]. Also, other studies suggested similar positive implications of cannabinoids from hemp in gastrointestinal mucosal defense, inflammation and oxidative stress [89,90].

The second step of this study was to assess the effect of the hemp diet on the oxidative status of piglets. Stressful events like weaning play a major negative role in modern livestock production [91]. It has been observed that if the diet of sows is rich in bioactive compounds, piglets better resist the challenges of the weaning period, such as transitory inflammation [62]. To the best of our knowledge, this is the first study concerning the effect of hemp seeds as dietary late gestation and early lactation intervention on the oxidative status of sows and suckling piglets. Our results showed that the plasma oxidative status of piglets derived from sows fed HD decreased below that of the control in all the three measurement time points of the suckling period, except for GPx activity on day 1. Similar to the sows, the significant reduction in TBARS levels in conjunction with the mitigating tendency for ROS in piglets as well as the increase in TAC levels implies an adequate antioxidant status. Hence, PUFAs might be protected against peroxidation, leading to increased generation and bioavailability of nitric oxide [92]. Indeed, we found a significant prominent increase in NO production for piglets derived from sows fed HD compared to the control group, suggesting the efficacy of dietary hemp in the diet of sows. This might be beneficial for piglets taking into account the antimicrobial role of NO [60,61]. In particular, the difference was higher on day 1, and much smaller on days 7 and 21. The antioxidant properties of bioactive compounds (PUFAs, cannabinoids, etc.), especially when a low ω-6:ω-3 PUFA ratio is present, are related to the activation of Nuclear factor (erythroid-derived 2)-like 2 (Nrf2), which plays a key role in the protection against oxidative stress [83,93]. Also, the activation of cannabinoid receptor type II (CB2 receptor) showed beneficial potential in mitigating oxidative stress and inflammation in several disease models [94,95,96].

In our experiment, the comparison between the piglets derived from sows fed either HD or CD, revealed a significant diet effect on antioxidant enzyme activities. SOD activity in HD piglets was higher overall than CD (steady levels), showing a decreasing trend towards the end of the suckling period. A notable aspect is the magnitude of enzyme activity for SOD and CAT in the first day of lactation in piglets derived from HD sows in comparison with the control group which might be a consequence of feeding hemp during gestation. Elevated SOD activity and total antioxidant capacity was also noticed by Avramovic et al. (2012) and Lionetti et al. (2012) as an effect of dietary ω-3 fatty acid supplementation [97,98]. Hemp diet positively affected the activity of catalase over the entire lactating period. Similarly, we found that time effect was significant for GPx activity. Even though the levels were lower on day 1 for HD piglets, the increase over time was greater than CD as indicated by the significant D × T interaction. This variation in GPx was also reported elsewhere [99], showing low concentrations in piglets at birth, followed by a large increase during the first week of age (7 days). A similar profile with SOD and CAT was observed for total antioxidant capacity, which increased significantly in the plasma of piglets suckling from sows fed hemp. HD also influenced ROS and TBARS concentrations, but no D × T interaction was observed, which delineates the similar parallel trends for the two diets. In association with TAC, the overall improvement in TBARS and the tendency to lower ROS, may indicate the potential of HS to mitigate the action of free radicals.

As suggested so far, the most important of our findings is the link between the sows and progeny, evidenced by the similar trend in NO synthesis and GPx activity over the entire experimental period. On the other hand, we observed that ROS was not influenced by hemp, neither in sows nor in piglets over time. In piglets, we observed a decrease in ROS levels, while, in sows, there was no difference in ROS evolution over time. Also, TBARS was the highest on day 1 in sows, while it rose over time in piglets. Nevertheless, the inclusion of HS in the diet of sows had an overall positive influence on the antioxidant status markers in piglets.

Finally, future research would be helpful to understand the effects of dietary hemp seeds in terms of the most efficient moment of intervention.

### Study Limitations

The present experimental approach had some limitations. Firstly, the experiment was done in a small experimental farm employing the rationale of maximizing animal utilization. The small number of piglets used herein (only 16) resulted from a subsequent assignment of the rest of the progeny to other dietary hemp inclusion rates during growing and finishing-fattening periods. Also, data from four piglets were discarded due to unavailable blood samples at day 21. 

Aside from the fact that the piglets in our study had ad libitum access to the solid feed, their main nutrient source was the milk suckled from the sows. In this case, their intake is reflected through the reported average daily gain (ADG).

From a statistical point of view, linear, quadratic and cubic polynomials analysis would have been considered but, because the study design involved the same sows and litters and measurements at different time points, the repeated measurements was chosen as the best for a relevant and most appropriate statistical approach.

Another study limitation of this research was the use of TBARS as an indicator of lipid peroxidation, while other alternatives might be available, such as the potential use of the oxidant status index (pro-oxidants/antioxidants). There are many controversies as to whether TBARS is appropriate for assessing oxidative status as TBARS is not specific for MDA and MDA itself represents an end product of lipid peroxidation. An ultra-high-performance liquid chromatography (UHPLC) method for MDA determination would be more appropriate, but this is still being set up in our laboratory. However, additional parameters (i.e., ROS, TAC) were measured in conjunction with TBARS. The TBARS test is perceived as an important indicator of oxidative status. 

The study lacks the assessment of an additional treatment group fed a diet containing all the constituents of hemp excluding the polyunsaturated fatty acids (PUFAs). Nevertheless, the observation that hemp seed was able to improve the overall oxidative status markers in sows and their offspring indicates that hemp seed is an efficient alternative dietary approach.

## 5. Conclusions

Our study revealed the positive effect of hemp diet on antioxidant enzyme activities together with increased TAC and NO production, as well as an improvement in TBARS levels in sows. Similarly, the elevated state of antioxidant defense system and increase in NO production in relationship with the low TBARS levels during lactation might lead to a better response of piglets receiving HD to weaning. This hypothesis deserves further investigation as to whether they could be correlated. The descriptive findings on plasma levels could be perceived as increased adaptability of piglets towards maintaining an increased antioxidative systemic status with regard to the upcoming challenge of weaning. Collectively, the results obtained in this study provide new insight into the beneficial effects of the dietary addition of hemp seeds as early life intervention. 

## Figures and Tables

**Table 1:** Total polyphenol content and antiradical activity of hemp seed and experimental diets.

Sample	Analysis			
	TPC (mg GAE/100 g Raw Material)		A_AR_ (µM TE)	
	Mean	*SEM*	Mean	*SEM*
HS	1000.05 ^b^	7.5	467.63 ^b^	3.4
CD	1178.04 ^a^	4.1	517.17 ^a^	4.9
HD (5%)	1146.66 ^a^	5.5	513.83 ^a^	10

TPC = total polyphenol content; A_AR_ = antiradical activity measured using DPPH radical; GAE = gallic acid equivalents; TE = Trolox equivalents; HS = hemp seeds; CD = control diet; HD = hemp diet; SEM = standard error of the mean. All values are represented as means with their standard errors. ANOVA (one-way) was performed followed by Student’s *t* test; ^a,b^ = Mean values within a column with different superscript letters were significantly different (*p* < 0.05).
